# Significance of PET-CT for Detecting Occult Lymph Node Metastasis and Affecting Prognosis in Early-Stage Tongue Squamous Cell Carcinoma

**DOI:** 10.3389/fonc.2020.00386

**Published:** 2020-04-09

**Authors:** Guo Zhao, Jianli Sun, Kai Ba, Yunxiang Zhang

**Affiliations:** ^1^Department of Oral Medicine, The First Affiliated Hospital of Zhengzhou University, Zhengzhou, China; ^2^Department of Endodontics, Kaifeng Stomatology Hospital, Kaifeng, China

**Keywords:** PET-CT, occult lymph node metastasis, early-stage tongue squamous cell carcinoma, tongue squamous cell carcinoma, oral squamous cell carcinoma

## Abstract

**Objective:** We aimed to clarify the significance of PET-CT for detecting occult lymph node metastasis and for affecting prognosis in early-stage tongue squamous cell carcinoma (SCC).

**Methods:** Patients with surgically treated primary cT1-2N0 tongue SCC who agreed to undergo a preoperative PET-CT scan were prospectively enrolled. The primary study outcomes were occult neck lymph node metastasis and locoregional control (LRC). The Kaplan-Meier method was used to analyze the LRC rate, and then the factors that were significant in the Kaplan-Meier method were assessed in the Cox model to determine the independent factors.

**Results:** A total of 135 patients were included, and the median maximum standardized uptake value (SUV max) of the primary tumor was 9.0. When analyzing the PET-CT results, 18 patients were recognized as having neck lymph node metastasis, and 12 patients were proven to have pathologic lymph nodes. A total of 117 patients did not have neck lymph node metastasis reported by PET-CT, and five patients were proven to have pathologic lymph nodes. The sensitivity and specificity of PET-CT for predicting occult metastasis were 70.6 and 94.9%, respectively. In patients with an SUV max ≤ 9.0, the 5-year LRC rate was 95%; in patients with an SUV max >9.0, the 5-year LRC rate was 85%, and the difference was significant. Further Cox model analyses confirmed the independence of the SUV max for predicting LRC.

**Conclusion:** PET-CT has a high specificity for predicting occult lymph node metastasis, and an SUV max >9.0 is significantly associated with worse LRC in cT1-2N0 tongue SCC.

## Introduction

Tongue squamous cell carcinoma (SCC) is a common malignancy in the oral cavity, and complete resection is the standard treatment procedure (1, 2). Owing to the wide range of occult lymph node metastasis rates, there is controversy regarding the best neck management. It is important for us to detect patients who are at high risk of neck lymph node metastasis preoperatively. Current researchers have demonstrated the role of perineural invasion, lymphovascular invasion, depth of invasion, neutrophil-to-lymphocyte ratio, and so on in predicting occult lymph node metastasis (3–6), but the significance of PET-CT for determining occult lymph node metastasis has rarely been discussed. Zhang et al. (7) described that the overall sensitivity and specificity of PET-CT in cT1-2 oral SCC were 21.4 and 98.4%, respectively, with a negative predictive value of 99.1%. Myers et al. (8) demonstrated an estimated overall sensitivity of PET-CT for the N0 neck of 78% with a specificity of 100%. On the other hand, cancer cells use glucose as an energy source, and tumors with strong growth potential and invasiveness are highly likely to take up FDG (9), as indicated by the maximum standardized uptake value (SUV max). Previous authors have depicted the prognostic role of the SUV max in head and neck SCC; however, all these studies included all subsites or analyzed large-volume tumors together with small-volume tumors. Considering that different biological behaviors exist between tongue SCC and SCC at other sites, in the current study, we aimed to clarify the significance of PET-CT for detecting occult lymph node metastasis and affecting prognosis in early-stage tongue squamous cell carcinoma.

## Patients and Methods

The Zhengzhou University Institutional Research Committee approved our study (No. 201845YZ), and all patients signed informed consent agreements for medical research before the initial treatment. All methods were performed in accordance with the relevant guidelines and regulations.

From January 2010 to December 2018, consecutive patients with primary early stage (cT1-2N0) tongue SCC were prospectively enrolled. The only inclusion criterion was that the patient agreed to undergo a PET-CT examination preoperatively. Clinical pathologic and follow-up data, including age, sex, smoking status, drinking status, pathologic TNM stage based on the AJCC 8th edition, perineural invasion (PNI), lymphovascular invasion (LVI), extracapsular extension (ECS), depth of invasion (DOI), and SUV max of the primary tumor, were recorded in the enrolled patients.

Smokers/drinkers were defined as patients who smoked/drank at diagnosis or who had stopped for <1-year (10, 11). All pathologic sections were reviewed by at least two head and neck pathologists. PNI was considered to be present if tumor cells were identified within the perineural space and/or nerve bundle; LVI was positive if tumors were noted within the lymphovascular channels (12). The pathologic DOI was measured from the level of the adjacent normal mucosa to the deepest point of tumor infiltration, regardless of the presence or absence of ulceration (13). cT1-2 was defined by a maximum tumor diameter of <2 cm or ranging from 2 to 4 cm. Patients were considered cN0 if they had no evidence of nodal metastasis on a clinical exam, ultrasound, or radiographic imaging (not including PET-CT) (9). The cut-off value of the SUV max was set according to the median value (14).

Several PET/CT scanners were used to perform the PET-CT scans (GE Healthcare, Milwaukee, America). Patients fasted for at least 6 h before the PET-CT scan. The procedure was postponed when glucose levels were >200 mg/dL. Each patient received 10–20 mCi of [^18^F] FDG dose, based on his or her weight. Axial PET and diagnostic CT images were obtained from the calvarial vertex through the upper thighs after urinary voiding. Emission images were obtained after a radiopharmaceutical injection 1 h later. There was no contrast medium used during the CT scan. The images were reconstructed in the thickness of a 2.5 mm slice. The SUV max was measured for both the primary tumor and regional lymph nodes. For every suspicious lesion, the isocontour region of interest centered on the maximum value pixel was drawn automatically with workstation tools generating the SUV max of the region. An SUV max cut-off of 2.5 MBq/g was used for FDG-avid lymph nodes and primary tumors on PET-CT.

In our cancer center, systemic examinations, including ultrasound, CT, and MRI, were routinely performed. PET-CT was selectively suggested, and complete resection of the primary tumor with a margin of at least 1 cm, including the adipose tissue in the mouth floor as well as the sublingual gland and neck dissection (level 1–3), was routinely performed in every patient with any stage of tongue SCC. Adjuvant treatment was suggested if there was neck lymph node metastasis, PNI, LVI, or a positive margin. All patients were regularly followed up with every 3 months within the first 2- years after the operation and every 6 months within the third to fifth years after the operation. If there was suspicion of disease recurrence, active interference was performed.

The primary study outcomes were occult neck lymph node metastasis and locoregional control (LRC). The 4-fold table method was used to analyze the association between PET-CT and occult lymph node metastasis. The survival time was calculated from the date of surgery to the date of the first event of local, regional, or locoregional recurrence or to the latest follow-up. The Kaplan-Meier method was used to analyze the LRC rate, and then the factors that were significant in the Kaplan-Meier method were assessed in the Cox model to determine the independent factors. All statistical analyses were performed using SPSS 20.0, all reported *p*-value was two-sided, and *p* < 0.05 was considered to be significant.

## Results

There were 135 patients (109 males and 26 females) enrolled in total, and the mean age was 54.5 (range: 30–76) years. There were 76 (56.3%) smokers and 55 (40.7%) drinkers. The median SUV max was 9.0 (range: 2.3–29.7). Clinical tumor stages were characterized as cT1 in 57 (42.2%) cases and cT2 in 78 (57.8%) cases. 5 cT1 tumors were corrected into pT2 tumors after operation. PNI and LVI were noted in 18 (13.3%) and 15 (11.1%) patients, respectively. The mean DOI was 6.0 (range: 1.0–9.5) mm. Tumor differentiation was well in 53 (39.3%) cases, moderate in 70 (51.9%) cases, and poor in 12 (8.9%) cases. Negative margins were achieved in all patients.

Occult neck lymph node metastasis was reported in five of the 57 patients with cT1 disease with a rate of 8.8% and in 12 of the 78 patients with cT2 disease with a rate of 15.4%. ECS was reported in three (3/17, 17.6%) patients. The total number of positive lymph nodes was 23.

When analyzing the PET-CT results, 18 patients were recognized as having neck lymph node metastasis, and 12 patients were proven to have pathologic lymph nodes. A total of 117 patients did not have neck lymph node metastasis reported by PET-CT, and five patients were proven to have pathologic lymph nodes. The sensitivity and specificity of PET-CT for predicting occult metastasis were 70.6 and 94.9%, respectively.

**Figure 1:**
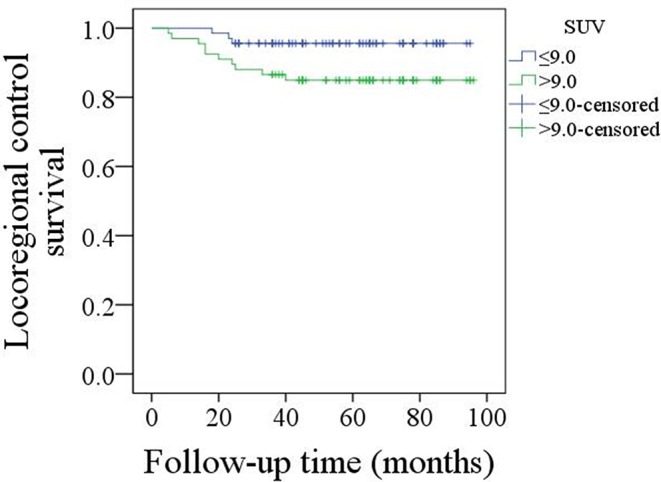
Comparison of locoregional control survival in patients with an SUV max ≤ 9.0 or >9.0 (*p* = 0.043).

The mean follow-up time was 53.9 (range: 6–95) months. Twenty five patients underwent adjuvant radiotherapy, while three patients also received adjuvant chemotherapy. Thirteen patients suffered from disease recurrence: four cases locally, five cases regionally, and four cases locoregionally. Eight patients were successfully salvaged with surgical treatment. In patients with an SUV max ≤ 9.0, there were three cases of recurrence, and the 5-year LRC rate was 95%. In patients with an SUV max >9.0, there were ten cases of recurrence, and the 5-year LRC rate was 85%; the difference was significant (Figure 1, *p* = 0.043). Further Cox model analyses confirmed the independence of the SUV max for predicting LRC (Table 1).

**Table 1:** Univariate and Cox model analyses of risk factors for the locoregional control survival in patients with cT1-2N0 tongue squamous cell carcinoma.

**Variables**	**Univariate**	**Cox model**	
	**Log-rank test**	***p***	**HR [95% CI]**
Age (<40 vs. ≥40)	0.458		
Gender	0.336		
Smoking	0.478		
Drinking	0.221		
SUV max (≤ 9.0 vs. >9.0)	0.043	0.034	2.445 [1.226–4.998]
Pathologic tumor stage	0.012	<0.001	3.668 [1.479–9.336]
Pathologic neck lymph node stage	0.024	0.421	4.612 [0.741–9.667]
Perineural invasion	0.089		
Extracapsular extension	0.642		
Lymphovascular invasion	0.031	0.042	2.889 [1.365–4.416]
Tumor differentiation	0.009		
Well			
Moderate		0.097	2.579 [0.947–5.667]
Poor		0.016	3.978 [1.741–8.665]
Radiotherapy	0.116		

## Discussion

The most important finding in the current study was that PET-CT had a high specificity for predicting occult lymph node metastasis, and an SUV max >9.0 was significantly associated with worse LRC in patients with early-stage tongue SCC. These results can provide better guidance regarding decision making in terms of neck dissection and can help determine which patients would need adjuvant radiotherapy.

Neck lymph node metastasis is the most important prognostic factor in oral SCC, and neck management for early-stage tongue SCC is a key issue. A few authors who support routine neck dissection depicted that it could be beneficial for accurately staging the neck and identifying patients who require adjuvant therapy as well as improving survival (15). However, some authors have reported that the rate of occult neck lymph node metastasis varies, and a considerable number of patients have been over-treated (16). This controversy prompted the effort to determine the possible predictors for occult lymph node metastasis. Larson et al. (17) aimed to clarify the contribution of adverse pathologic characteristics to clinical outcomes in small tongue SCC and concluded that, even though PNI and LVI occurred, the prevalence of occult lymph node metastasis was very low in small disease with a DOI ≤ 4.0 mm. Wu et al. (18) described that a DOI larger than 4 mm and a stable growth pattern in the invasive front were independent risk factors for occult lymph node metastasis in cT1-2N0 tongue SCC. A similar finding was also reported by Faisal et al. (19): 179 patients with early-stage tongue SCC were divided into three groups according to the AJCC cutoff points in the 8th edition according to depth(group A: 1–5 mm, group B: 6–10 mm, and group C: > 10 mm). The authors noted that the risk of local recurrence and nodal metastasis in group A was 15 and 23%, in group B was 20 and 34%, and in group C was 40 and 53%, respectively. However, all these studies were retrospective, and the analyzed predictors, including DOI, PNI, and LVI, usually remained unknown during the frozen section, which would greatly limit their clinical application.

Cancer cells utilize glucose as energy in general, and PET-CT has been widely used to detect the primary site and metastasized lymph nodes. Additionally, a number of researchers have clarified the role of PET-CT in evaluating neck status. Chaukar et al. (20) previously compared the diagnostic accuracy of staging a cN0 neck among ultrasound, contrast enhanced CT, and PET-CT in 70 oral SCC patients. The authors reported that the PET-CT scan had a poor specificity of 54.1% owing to the high false-positive rate, and the CT scan showed the best accuracy with 80.2% with a specificity of 85.4% and sensitivity of 73.6%. The authors concluded this strange finding was contributed in part by the epidemic of chronic granulomatous diseases in India. Currently, more and more researchers are describing a convincing result. Bae et al. (21) enrolled 178 oral SCC patients with negative neck palpation findings, and found the sensitivity for the detection of occult metastasis was higher for PET-CT than that for CT/MRI imaging on a per-patient (69.1% vs. 35.7%), per-level (62.1% vs. 29.3%), and per-side (70.5% vs. 36.4%) basis. Gordin et al. (22) found that PET-CT had a sensitivity of 89% and a specificity of 95% for predicting neck lymph node metastasis in head and neck SCC. A similar finding was also reported by Jeong et al. (23) and Roh et al. (24); however, all these studies included SCCs of all subsites, and small-volume cancers were analyzed together with large-volume cancers. The prevalence of neck lymph node metastasis is significantly associated with tumor stage, but the role of PET-CT in early-stage tongue SCC has rarely been discussed. Zhang et al. (7) might have been the first to report that the overall sensitivity and specificity of PET-CT for predicting occult lymph node metastasis were 21.4 and 98.4%, respectively. This finding was partially consistent with our results. The high specificity found in both studies is possibly attributed to the low occult lymph node metastasis rate. The significant conflict of sensitivity between the two studies might be explained by the following aspects: the different economic status that exists between China and other Western countries might cause differences in oral hygiene, and it is well-known that infectious lymph nodes and inflammation lead to false-positive PET-CT outcomes. The findings of the two studies might suggest that if there is a negative result according to PET-CT, there may be a very high possibility of no occult lymph node metastasis. Some may argue, then, that active observation is an option for early-stage tongue SCC. There have been conflicting results regarding the comparison between elective neck dissection and active observation in cT1-2N0 tongue SCC (15, 16). Another uncommon tool that has been used is sentinel lymph node biopsy (SLNB). Recently, Cramer et al. (25) compared the survival difference between SLNB and elective neck dissection groups; the authors reported that neck dissection was avoided in 63.8% of patients receiving SLNB, the two groups had similar overall survival, and decreases in perioperative morbidity and hospital stay were seen in patients with SLNB. Alex et al. (26) described that SLNB was easily performed with a high success rate, and it had a relatively high specificity and a low false-negative rate. Similar findings were also reported by Holden et al. (27) and Den Toom et al. (28). Both SLNB and PET-CT had high value for predicting nodal metastasis in oral SCC. However, in China, SLNB is not widely used for oral SCC. Although the risk is low, SLNB is invasive. Klode et al. (29) noted that SLNB was much more sensitive than PET-CT for discovering small lymph node metastases in malignant melanoma; however, in cervical cancer patients, Papadia et al. (30) demonstrated that, compared to SLNB, PET-CT represented a “safety net” that helped the surgeon identify metastatic lymph nodes, especially in patients with large tumors. On the other hand, even though our unpublished data showed that PET-CT-guided neck dissection could achieve similar disease control to routine neck dissection, the cost utility as well as the actual applicability of PET-CT remains unknown, and further studies are needed to clarify these questions.

The prognosis of tongue SCC has been frequently analyzed. The widely accepted prognostic factors include tumor stage, neck lymph node stage, PNI, LVI, margin status, and so on (1–3, 5). However, the significance of the SUV max remains unclear. The SUV max is partially related to the malignant grade of tumor cells, reflecting proliferation ability. Hasegawa et al. (31) described that an SUV max >8.0 indicated a higher tumor stage, neck lymph node metastasis, the presence of PNI and LVI, and a higher Ki-67 index. Further survival analysis also confirmed that worse disease-free survival was indicated by an SUV max >8.0. Yokobori et al. (32) reported that patients with T2 stage disease had a higher SUV max than patients with T1 stage disease, and patients with a higher SUV max tended to have a higher frequency of PNI, microvessel density, and expression of LAT1. This finding was consistent with our results, suggesting that the SUV max might be used as a marker of adjuvant treatment in future studies.

The main limitation in the current study might be that it seems to provide the questionable message that PET-CT is suitable for all patients with low-stage oral tongue SCC, which slightly contradicts most recently published guidelines; however, what we wish to demonstrate is that PET-CT is an alternative in select patients. It is also a reliable method for guiding cN0 neck management in early-stage tongue squamous cell carcinoma.

In summary, PET-CT has a high specificity for predicting occult lymph node metastasis, and an SUV max >9.0 is significantly associated with worse LRC in cT1-2N0 tongue SCC.

## Data Availability Statement

All data generated or analyzed during this study are included in this published article, and the primary data can be provided by the corresponding author.

## Ethics Statement

The studies involving human participants were reviewed and approved by The Zhengzhou University institutional research committee. The patients/participants provided their written informed consent to participate in this study.

## Author Contributions

All the authors contributed to study design, manuscript writing, study selection, data analysis, study quality evaluating, and manuscript revising. All authors have read and approved the final manuscript.

### Conflict of Interest

The authors declare that the research was conducted in the absence of any commercial or financial relationships that could be construed as a potential conflict of interest.
